# Supplementary material for: Construction of Polypyrrole-Coated CoSe2 Composite Material for Lithium-Sulfur Battery
Source: Nanomaterials (Basel). 2023 Feb 25;13(5):865. doi: 10.3390/nano13050865 (PMC10005037; doi:10.3390/nano13050865)
Supplement: Supplementary file 1 [file nanomaterials-13-00865-s001.zip › nanomaterials-2226659-supplementary.pdf]

nanomaterials

Supplementary Materials

Construction of Polypyrrole-Coated $CoSe_2$ Composite Material for Lithium-Sulfur Battery

Yinbo Wu [1,*], Yaowei Feng [2], Xiulian Qiu [2], Fengming Ren [2], Jian Cen [1], Qingdian Chong [2], Ye Tian [2] and Wei Yang [2,*]

[1] School of Automation, Guangdong Polytechnic Normal University, Guangzhou 510665, China
[2] School of Chemistry and Chemical Engineering, GuangZhou University, GuangZhou 510006, China
* Correspondence: gdin_wyb@gpnu.edu.cn (Y.W.);

1. Experiment

1.1. Preparation of ZIF-67 and hollow $CoSe_2$ samples

The $CoSe_2$ samples were prepared by the combination of co-precipitation and high-temperature carbonization. First of all, according to the stoichiometric ratio, 8mmoL cobalt nitrate ($Co(NO_3)_2{\cdot}6H_2O$, AR, Shanghai Maclin Biochemical Technology Co., LTD) and 32mmoL 2-methylimidazole (AR, Shanghai Maclin Biochemical Technology Co., LTD, Shanghai, China) were added to beaker containing 100mL methanol solution (99.9%, AR, Aladdin Chemistry Co., Ltd , Shanghai, China) respectively, stirring until dissolved. The two solutions were mixed and stirred for 30min, and then left to rest at room temperature for 24h. The purple precipitation were washed by methanol, centrifuged and dried at 80 ° C for 12h to get purple powder ZIF-67. ZIF-67 and selenium powder (AR, Aladdin Chemistry Co., Ltd , Shanghai, China) were placed in a crucible with a certain mass ratio, calcined at 600° C for 3h in a tubular furnace in nitrogen atmosphere to get $CoSe_2$ samples.

1.2. Preparation of hollow $CoSe_2$@PPy dodecahedrons

First, 1.5 mmol sodium p-toluene sulfonate (p-TSS, AR, Shanghai Maclin Biochemical Technology Co., LTD, Shanghai, China) was added to 30 mL of mixed solution containing pure water/ethanol (v/v, 1:1), followed by 0.116 g of pyrrole monomer (99.9%, AR, Aladdin Chemistry Co., Ltd, Shanghai, China) and stirred until a uniform mixed solution A. At the same time, 3.75 mmol ammonium persulfate (APS, AR, Shanghai Maclin Biochemical Technology Co., LTD , Shanghai, China) as an oxidant, was added to 30 mL aqueous solution after intense agitation to gain solution B. Then place 0.1 g $CoSe_2$ powder into a clean beaker with 40mL DI and disperse it ultrasonic for 30 minutes, and then slowly drop solution A into the beaker. 30 minutes later, solution B was also dropped and polymerized under ice bath conditions. The sample was then placed in darkness for 24 hours and the residue was successively removed with pure water and methanol. At last, the black sample was dried overnight at 60 °C to gain $CoSe_2$ coated with in situ functional layers modified by PPy.

1.3. Preparation of hollow $CoSe_2$@PPy-S

The $CoSe_2$@PPy nanocomposites were mixed with S at 3:7 mass ratios and sealed in glass tubes. After being heated at 155 °C for 12 hours, the products were collected for characterization after cooling. $CoSe_2$-S material was prepared under the same conditions for comparison.

1.4. Measurement of material characteristics

The crystal phase of the sample was analyzed by XRD (PW3040/60, Netherlands). The morphological and structural characteristics of the prepared materials were investigated by SEM (JSM-7001F, Japan) and the lattice fringe of materials were tested by HRTEM (JEM-2100F , Japan). The PPy was further detected by FTIR (Spectrum100, Massachusetts, USA). The specific surface area and pore size of the cathode matrix materials were measured and analyzed by MEANS of ASAP 2460 BET specific surface analyzer. Sulfur mass was calculated by TGA (NETZSCH STA 449 F3/F5, Germany) in nitrogen at 10 °C min^{-1} from 30 to 600 °C. The chemical composition and valence of the composites were determined by XPS (Thermo Scientific K-Alpha, Massachusetts, USA). The adsorption performance of the composite material to lithium polysulfide was tested by UV-vis spectrometer (UV-3600, Japan).

1.5. Electrochemical characterization of materials

The positive electrode cut piece is made into a circular piece with a diameter of 12mm for use. Lithium plates (thickness of 2mm) were used for the negative electrode, Celgard2400 for the diaphragm, 1.0mol/L-1LiTFSI-DOL/DME (v/v, 1:1) -0.1M $LiNO_3$ for the electrolyte (40μL/per cell), and the test button battery (CR2032) was assembled in a glove box filled with argon. The resulting battery tests were performed on a charge-discharge meter at room temperature, alternating current impedance test and cyclic voltammetry (CV) tests were experimented at the CHI600 electrochemical workstation.

1.6. Visualization experiment test

Firstly, to form a concentration of 0.05M Li_2S_6 solution, Add the sulfur powder and lithium disulfide (molar ratio 5:1) to DOL/DME(v/v,1:1) solution and whisk together at 60°C for 48h until thoroughly combined to gain Li_2S_6 solution for reserve. Take 20mg of the active substance into 5ml of diluted (0.005M) Li_2S_6 solution and let it stand for 6h to observe the change in color of the solution. Then take the supernatant fluid for the UV-vis absorption spectrum test.

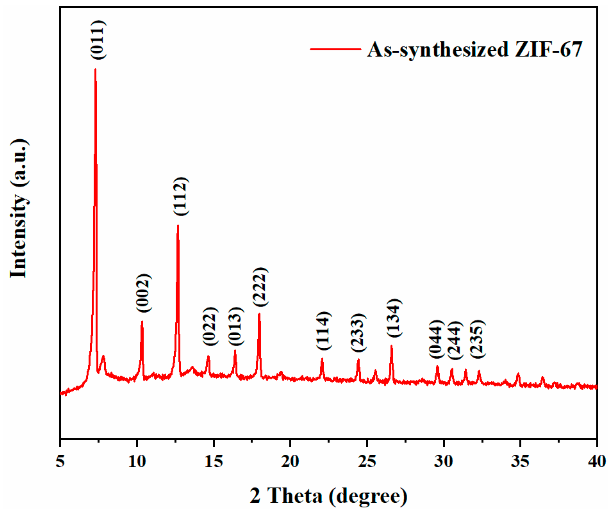

Figure S1. XRD patterns of ZIF-67 composites.

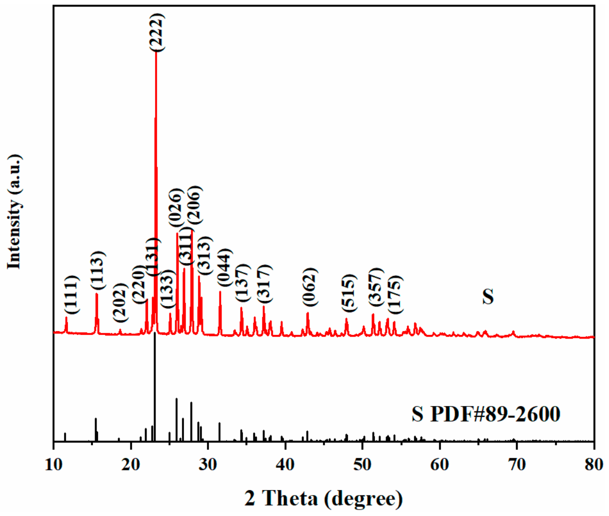

Figure S2. XRD patterns of Bare S.

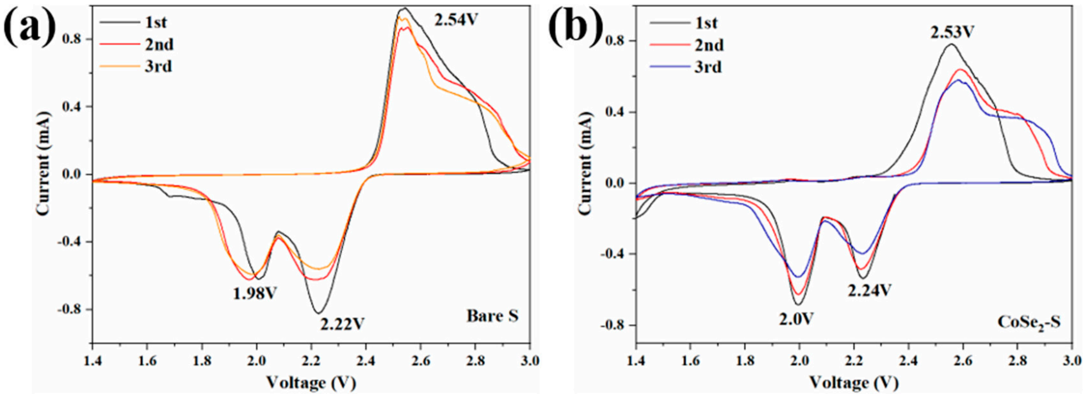

Figure S3. Initial three CV curves of (**a**) Bare S and (**b**) $CoSe_2$-S composite at a scan rate of 0.1 mV s^{-1}.

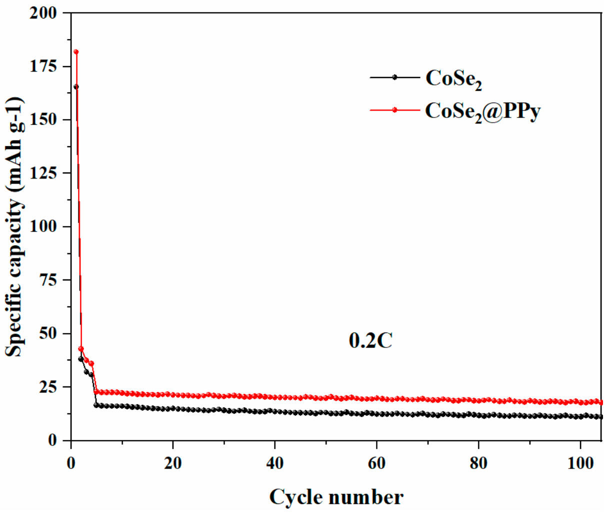

Figure S4. Cycling performance of $CoSe_2$ and $CoSe_2$@PPy at 0.2C over 100 cycles.

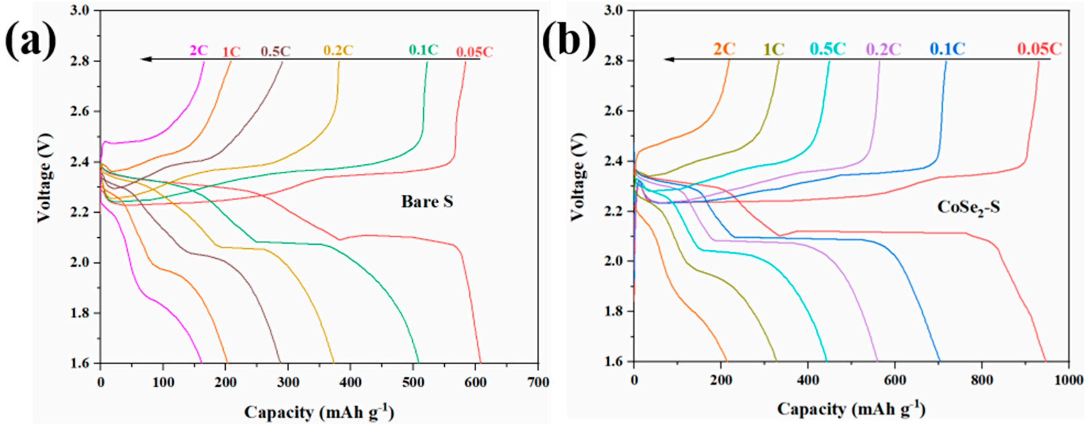

Figure S5. Galvanostatic discharge–charge voltage profiles of (**a**) bare sulfur and (**b**) $CoSe_2$ -S electrodes at different discharge/charge current rates.

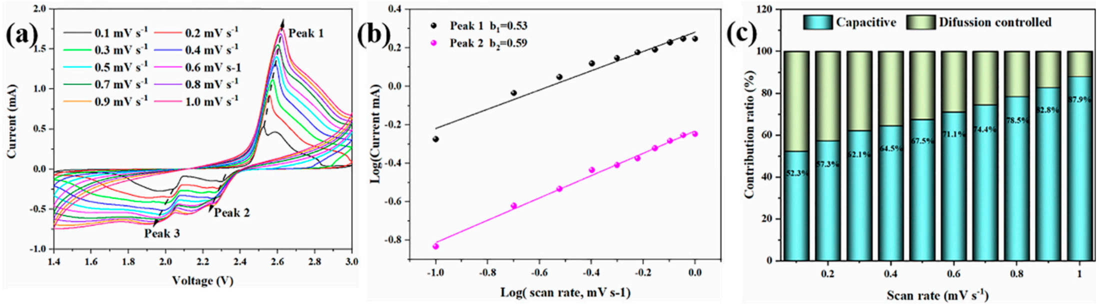

Figure S6. (**a**) CV curves of $CoSe_2$-S composites at different scan rates. (**b**) Profile of log(i) versus log(v) plots. (**c**) Charge contribution from capacitance and diffusion at various scan rates.
